# Mechanically Stable Ultrathin Layered Graphene Nanocomposites Alleviate Residual Interfacial Stresses: Implications for Nanoelectromechanical Systems

**DOI:** 10.1021/acsanm.2c03955

**Published:** 2022-12-14

**Authors:** Maxime Vassaux, Werner A. Müller, James L. Suter, Aravind Vijayaraghavan, Peter V. Coveney

**Affiliations:** † Université de Rennes, CNRS, IPR (Institut de Physique de Rennes), UMR 6251, Rennes 35000, France; ‡ Centre for Computational Science, Department of Chemistry, University College London, London WC1H 0AJ, United Kingdom; § Department of Materials and National Graphene Institute, The University of Manchester, Manchester M13 9PL, United Kingdom; ∥ Advanced Research Computing Centre, University College London, London WC1H 0AJ, United Kingdom; ⊥ Informatics Institute, University of Amsterdam, Amsterdam 1098 XH, The Netherlands

**Keywords:** electromechanical systems, ultrasonic transducers, graphene nanocomposite, molecular dynamics, interfacial stress, pressure sensors, polymer deposition

## Abstract

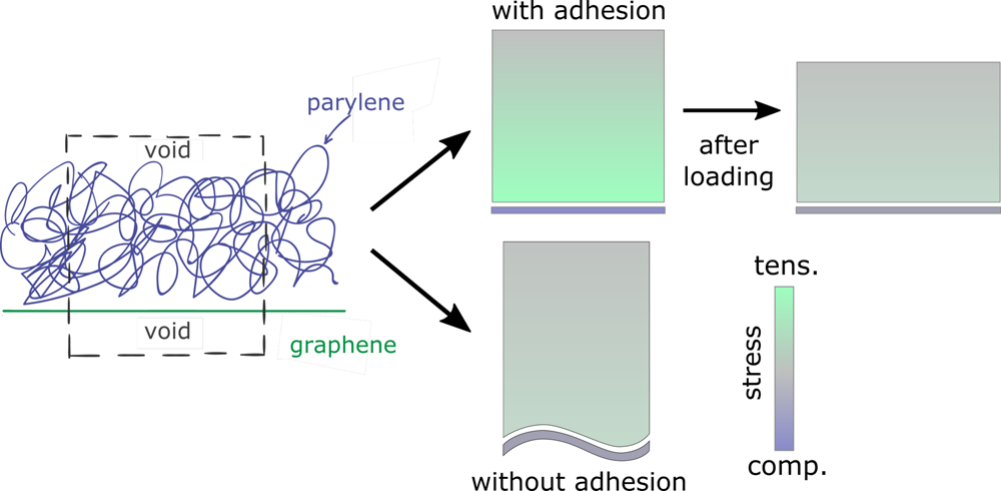

Advanced nanoelectromechanical systems made from polymer dielectrics deposited onto 2D-nanomaterials such as graphene are increasingly popular as pressure and touch sensors, resonant sensors, and capacitive micromachined ultrasound transducers (CMUTs). However, durability and accuracy of layered nanocomposites depend on the mechanical stability of the interface between polymer and graphene layers. Here we used molecular dynamics computer simulations to investigate the interface between a sheet of graphene and a layer of parylene-C thermoplastic polymer during large numbers of high-frequency (MHz) cycles of bending relevant to the operating regime. We find that important interfacial sliding occurs almost immediately in usage conditions, resulting in more than 2% expansion of the membrane, a detrimental mechanism which requires repeated calibration to maintain CMUTs accuracy. This irreversible mechanism is caused by relaxation of residual internal stresses in the nanocomposite bilayer, leading to the emergence of self-equilibrated tension in the polymer and compression in the graphene. It arises as a result of deposition–polymerization processing conditions. Our findings demonstrate the need for particular care to be exercised in overcoming initial expansion. The selection of appropriate materials chemistry including low electrostatic interactions will also be key to their successful application as durable and reliable devices.

## Introduction

**Figure 1:**
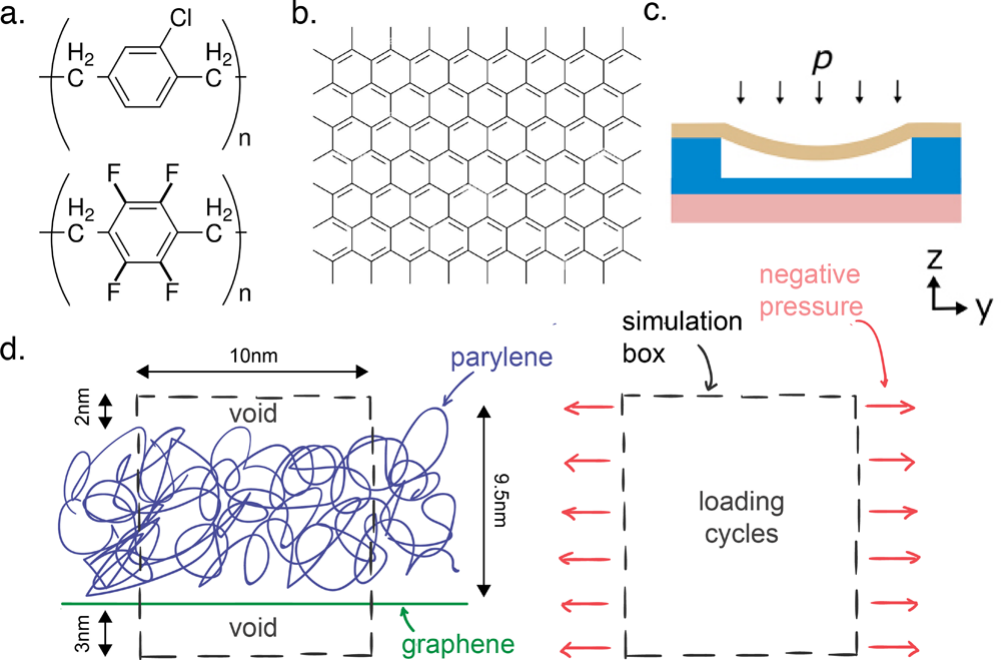
Materials, CMUTs, and membrane model. (a) Skeletal formula of parylene-C (top) and parylene-F (bottom) and (b) graphene. (c) Lateral view of the pressure sensor (yellow) global loading setup, supported (blue) only on the sides and bending under the application of vertical pressure *p*. (d) Schematization of the molecular membrane model (left) with periodic boundary conditions (dashed lines), where bending is applied on the molecular model as in-plane tension (red arrows) on the edges of the periodic box.

Graphene is regarded as an ideal actuating membrane for micro- and nanoelectromechanical systems (MEMS/NEMS) owing to its combination of superlative properties,^1^ the lowest known areal density, highest known elastic stiffness, lowest known bending modulus, and the highest known electrical and thermal conductivities of any material. A wide range of graphene-based NEMS devices have been demonstrated,^2^ including capacitive pressure sensors, microphones, capacitive micromachined ultrasound transducers (CMUTs), and accelerometers. Nevertheless there are some limitations encountered with graphene such as the low device yield due to damage or the contamination of graphene during chemical vapor deposition growth and transfer from the parent to the target substrate.^3^ Recently, this has been overcome by employing a laminated hybrid membrane comprised of graphene and an ultrathin polymer layer, resulting in 100% yield of high performance graphene NEMS pressure sensors^4^ and acoustic transducers.^5^ Ultrathin bilayer nanocomposites are therefore promising materials also for higher frequency devices such as CMUTs and resonant sensors. One of the polymers of choice, parylene-C, has also been demonstrated as an effective dielectric for graphene nanoelectronic devices such as field effect transistors^6^ and memristors.^7^ However, the mechanical stability of the combination of graphene and parylene-C (see Figure 1a,b) as ultrathin bilayer nanocomposites has not been investigated. This needs to be addressed now to guarantee sustainability, durability, and large-scale industrial adoption of these novel advanced materials.

For application of nanocomposite laminates as CMUTs, and more generally as NEMS, the mechanical properties of the biphasic nanomaterial must remain stable, that is, unchanged for a given loading range. In the case of mechanical properties, the material must remain elastic. Inelasticity of the nanocomposite mechanics causes sensor measurements drift and resolution issues and opens questions as to how often resulting additional calibration steps are required. One of the main causes of mechanical irreversibility is the degradation of the interface between polymer and graphene layers. Of course, the polymer itself is likely to dissipate mechanical energy under external loading; however, the interface constitutes the most likely location for slippage or void growth to occur. The mechanics of the polymer–graphene interface under external loading must therefore be investigated to ensure long-lasting calibration and durable application of layered nanocomposites.

The interface dynamics between highly homogeneous parylene-C and graphene materials mostly occur on the nanoscale. Indeed, the characteristic length of the interaction is tens of angstroms at most, depending on the charge distribution at the interface between graphene and parylene. The high-frequency loading associated with CMUT and resonant sensing applications renders the use of all-atom molecular dynamics (MD) simulations particularly suited. MD simulations provide access to local and high-resolution mechanical data at the interface typically inaccessible via characterization experiments only. We therefore make use of MD simulations to focus on the dynamics of a bilayer nanocomposite volume of approximately 1000 nm^3^ during the polymerization–deposition fabrication process and during operating conditions consisting of the application of transverse acoustic waves (see methods in Experimental Section). The simulations during operation follow the simulations of processing in order to replicate the mechanical state in which the nanocomposite is found during real-world application. We will perform MD simulations equipped with a standard nonreactive force field,^8−11^ as covalent bond breaking is not a significant mechanism of degradation for non-cross-linked polymers under operation conditions.

The accuracy of the prediction of our MD simulations is underpinned by their ability to reproduce parylene-C (see Figure S1), graphene,^12^ and graphene–polymer nanocomposite^9^ mechanical properties. Moreover, their statistical robustness is ensured by the use of ensembles constraining the intrinsic aleatoric uncertainty of MD.^13,14^

## Experimental Section

Classical molecular dynamics (MD) simulations were performed using the optimized potentials for liquid simulations (OPLS) force field,^15^ which we have already applied to several studies of graphene–polymer nanocomposites^8−11^ and has been shown to describe well fluorinated polymers.^16^ The OPLS force field features bonded (bond, angle, dihedral, improper) interactions as well as pairwise (van der Waals and Coulomb) interactions. The MD simulations were performed using the widely used LAMMPS code.^17,18^

### Monomers and 2D-Materials Description

MD simulations of deposition, polymerization, densification, equilibration, and loading are performed for several polymer nanocomposite formulations. The reference membrane in our study is assembled from a layer of parylene type C monomers deposited and polymerized onto an infinite graphene sheet (see Figure 1a,b). Parylene-C monomers are polymerized via covalent bonding of their terminal carbon atoms to form non-cross-linked chains of benzene rings. In order to investigate the effect of chemistry, we consider the case of parylene-F monomers, whereby the three hydrogen and one chlorine atoms on the benzene rings are replaced by four fluorine atoms (see Figure 1a). The graphene-based sheet models are generated using an open-source, verified and validated, 2D-materials builder (https://github.com/velocirobbie/make-graphitics/).^19^

### Simulation of High-Frequency (MHz) Cycles of Stretching

CMUT usage involves the application of orthogonal pressure to the mean plane of the bilayer nanocomposite membrane (see Figure 1c). Orthogonal pressure results in in-plane bending of the membrane. From a microscopic point of view, it is equivalent to the nanocomposite being stretched in-plane biaxially.

Sensing high-frequency pressure waves of MHz range means that the membranes face cycles of loading and unloading completed in the range of 10 ns or 1 μs. Further, as we want to observe the stability of the interface between the two constituents of the bilayer, we need to simulate enough cycles of loading for the irreversible mechanisms to occur. We simulated up to 50 loading cycles. Inherently, without any additional assumption, each individual replica MD simulation is used to compute the dynamics of the membranes for at least 100 ns to 1 ms.

However, we checked for potential separation of time scales in our molecular models of the membrane, which is often observed for thermoplastic polymer systems.^20^ We verified the convergence of the mechanical response of the material with decreasing strain rates. We actually determined that exactly the same variations of the three dimensions were found when applying in-plane stress to the membrane, whether it is applied over 10 ns or at least 100 ps (see Figure S2). Below 100 ps per cycle, i.e., at very high strain rates, elastic strains are not able to propagate in the system, overestimating its stiffness; we therefore observe underestimated strains during cycles of pressure controlled stretch. Based upon these observations, we assume that we can speed up our MD simulations 50-fold. Each cycle of loading and unloading is therefore applied in 200 ps instead of 10 ns.

### Deposition–Polymerization Process Simulation

**Figure 2:**
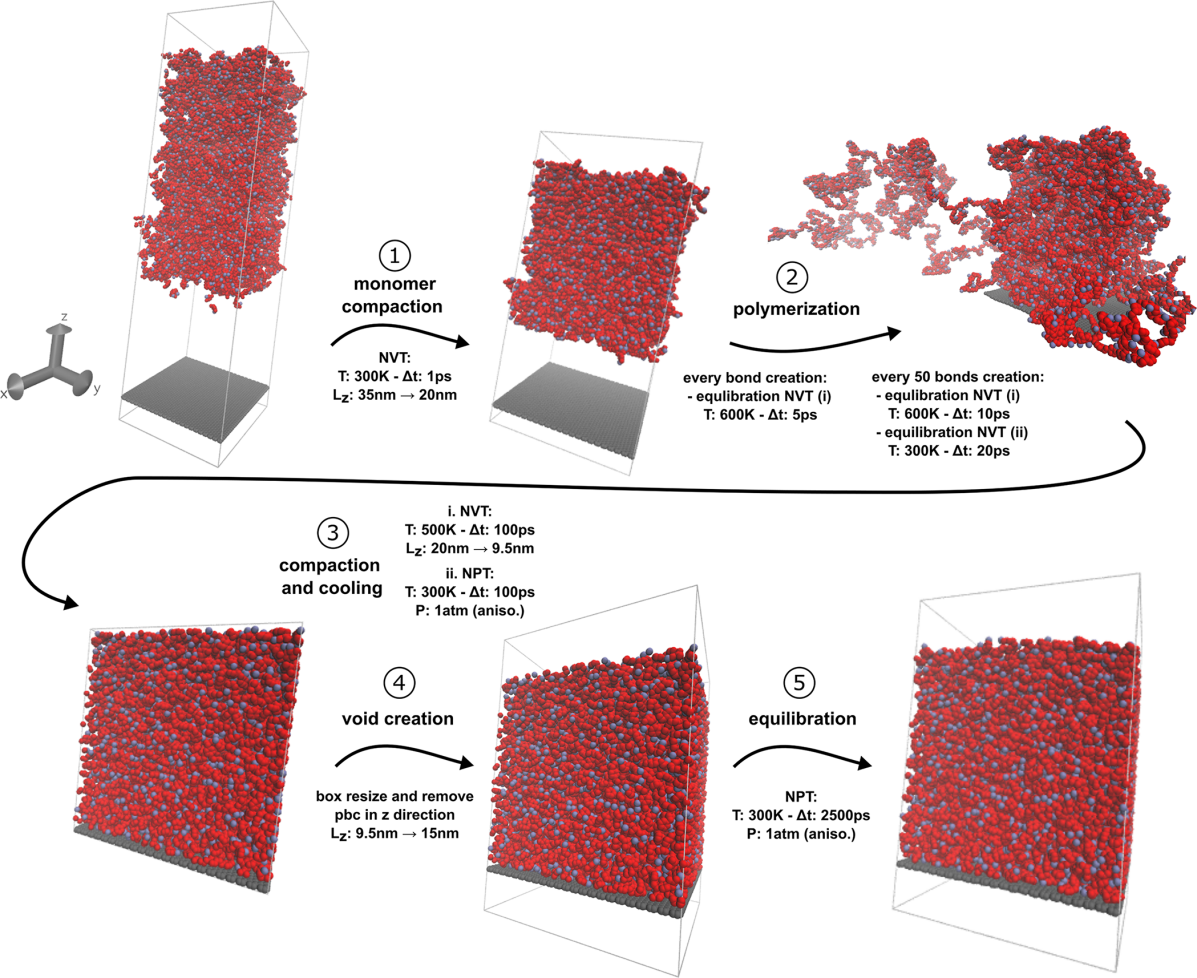
Simulation of the deposition–polymerization process. Monomers of parylene and graphene are inserted in an initial volume of 35 × 10 × 10 nm^3^. During step 1, the system is compressed vertically. In step 2, the monomers are linked together with covalent bonds to obtain the fully polymerized network. Cooling and compaction are applied in step 3 to build a polymer with industrial density. Voids are created in step 4, below and above the bilayer, to avoid interaction of the top surface of the polymer and the bottom surface of graphene. Finally, long equilibration of the system is simulated in step 5, to obtain the final system.

The processing procedure simulation aims at building molecular replicas of the semi-infinite bilayer nanocomposite in a periodic box of approximately 10 nm by 10 nm, in the in-place *x* and *y* dimensions. In the vertical dimension *z*, the system consists of one sheet of graphene superimposed with an approximately 10 nm thick layer of polymerized parylene-C (after compaction). The two contacting layers are surrounded by a 5 nm thick void (see Figure 1d). The deposition and polymerization procedure to obtain the final nanocomposite with desired material properties (e.g., density, glass transition temperature, and bulk/Young modulus) is performed in 6 stages (see Figure 2):

1. monomer packing;
2. bond-by-bond polymerization;
3. compaction;
4. cooling;
5. void creation;
6. equilibration.

In more detail the first step consists of randomly packing monomers of parylene within a large 15 × 15 × 15 nm^3^ volume using Polymatic (https://nanohub.org/resources/17278).^21^ The graphene sheet is inserted horizontally in a periodic box filled with monomers, parallel to the *x*–*y* plane. The resulting systems contain 5000 monomers of parylene, each containing 16 atoms and one graphene sheet of 3680 atoms, for a total of 83 680 atoms.

Polymerization is performed in the second step by covalent bonding of two terminal alkene carbons of different molecules, when found at a distance below 6 Å. The two candidate terminal carbons of all the monomers are initially enriched with a positive or negative electrostatic charge, respectively, to accelerate aggregation of pairs of candidate atoms. Polymerization is performed one bond at a time, for the closest pair of alkene carbons. Additional electrostatic charges are removed for newly bound atom pairs. Every 50 bonds formed, the system is briefly equilibrated and relaxed by means of a 1 ps long, constant volume and temperature (*NVT*), MD simulation at 600 K. For every 250 bonds formed, a longer relaxation of the system is simulated, at constant temperature and pressure (*NPT*), for 10 ps at 1 bar and 300 K. Polymerization is terminated when no more pairs of terminal carbons are found (<6 Å) and less than 1% of terminal carbons is left unbonded. At this stage, the additional electrostatic charges on terminal carbons are removed. The polymerization workflow is also facilitated by the use of the Polymatic software. During all our MD simulations, the time step is 1 fs and the damping parameter for temperature and pressure is 100 fs.

The nanocomposite system is then compressed vertically in the *z* dimension during the third step of the process. The thickness of the parylene layer is shrunk down to approximately 9.5 Å to reach the desired industrial density of 1.289 g/cm^3^. Densification is done above the glass transition temperature at 500 K. Immediately after, in the fourth step, the bilayer membrane is cooled from the compaction temperature down to room temperature (300 K). Both the third and fourth steps are performed using *NVT* MD simulations of 100 ps each.

An additional 5.5 nm void is inserted artificially between the bottom face of the graphene sheet and the top of the parylene layer, during the fifth step. This is done so as to avoid self-interaction of the nanocomposite through the periodic boundary conditions in the *z* dimension. Finally, the nanocomposite membrane is equilibrated for 2.5 ns by means of a constant pressure and temperature MD simulation, at 1 bar and 300 K, to enable the relaxation of newly formed polymer chains. The membrane is then assumed to be at equilibrium since the potential energy of graphene and parylene-C and the dimensions of the system do not vary significantly anymore (see Figure S3a,b).

With this deposition–polymerization process, we generate one replica of our nanocomposite molecular model. Different replicas are generated by the same process and differ only by the choice of the random seed. In turn, for each nanocomposite chemical formulation, we simulate ensembles of replicas of our molecular model in order to produce robust statistics. We determined in earlier studies of material properties predictions using MD simulations that with highly homogeneous systems such as polymers, aleatoric uncertainty can be contained with only a small number of replicas, as low as 5.^13,20^

## Results and Discussion

### Irreversible Dynamics at the Polymer–Graphene Interface

**Figure 3:**
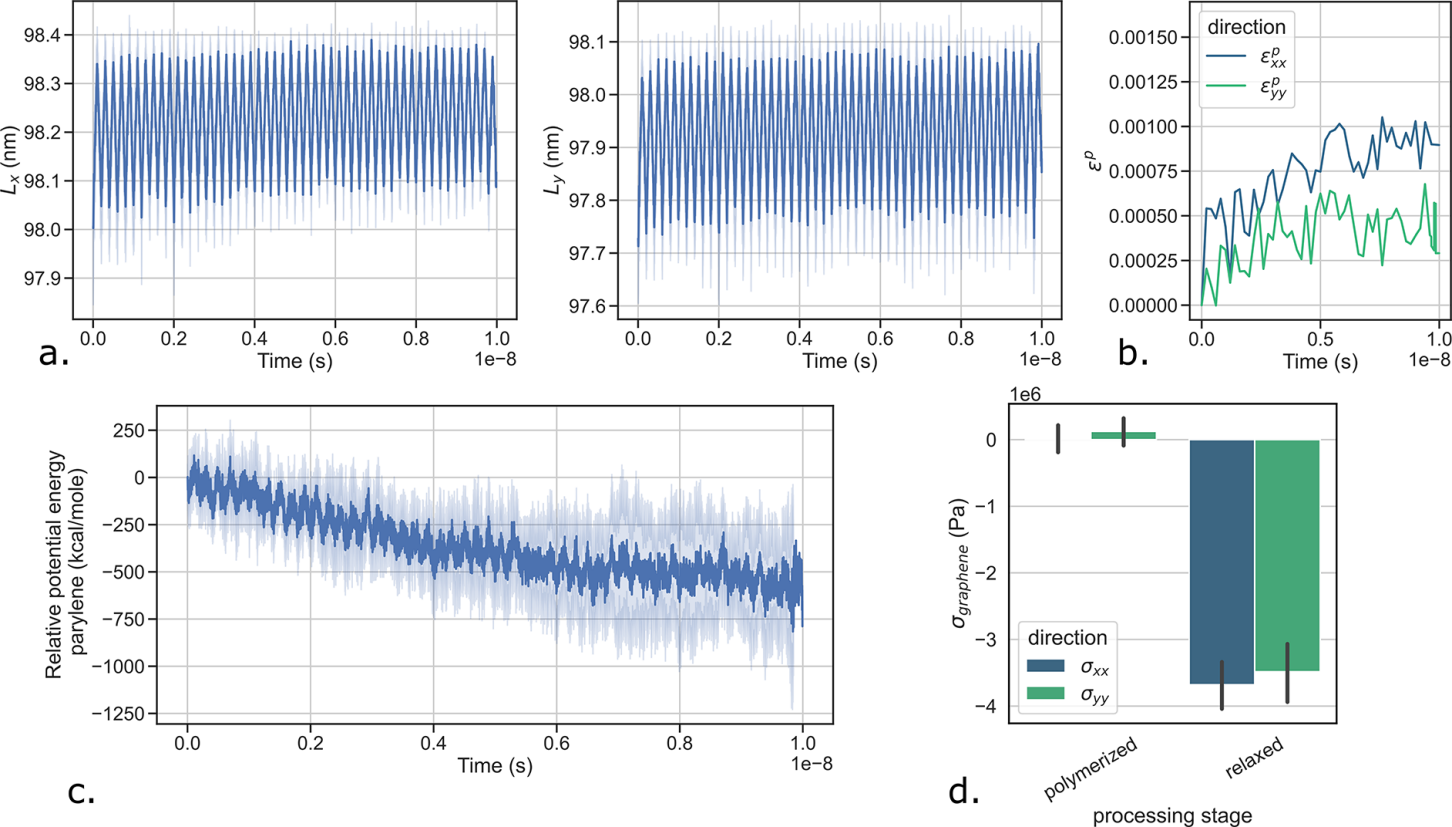
Permanent strain accumulation induced by residual stress relaxation. (a) Time evolution of the in-plane dimensions of the molecular model of the membrane during 50 cycles of external tension loading. (b) Accumulation of in-plane permanent strains ϵ_*xx*,*yy*_^*p*^ throughout the 50 cycles of loading. (c) Time evolution of the variation of the potential energy of the parylene layer (with respect to the initial potential energy), showing oscillations in phase with the loading and a continuous decrease. (d) In-plane compression stresses build up in the graphene sheet during the processing stages following the polymerization step. One standard deviation intervals from the ensemble-averaged predictions are displayed in light blue (a, c) and error bars (d).

We consider the relevant loading range of CMUTs to be transverse pressure-controlled cycles of loading associated with acoustic waves impacting the membrane (see methods in Experimental Section). At the molecular level this can be modeled by resulting in-plane cycles of stretching. We begin our investigation with the application of a small number of cycles of stretching (50 in total) to our ensemble of 5 replicas of bilayer nanocomposite membranes. Due to the high homogeneity of our systems, such small ensembles suffice, which is certainly not the case for most MD applications.^13,14^ We compute the evolution of the in-plane dimensions *L*_*x*_ and *L*_*y*_ of the membrane throughout the cycles of loading in response to the applied negative pressure (see Figure 3a). Real world applications of CMUTs tend to operate in a strain range of 0–1%. We therefore apply a linearly increasing pressure up to −80 MPa which results in a peak stretch in the ultrathin membrane of 1%. We observe that in both directions, but in particular in direction *y*, the initial dimension of the system is not recovered when unloading. The dimensions of the system at peak loading (−80 MPa) also increase. We quantify the accumulation of permanent axial strains ϵ_*xx*_^*p*^ and ϵ_*yy*_^*p*^ in both in-plane directions, which are the remaining strains at zero-loading (see Figure 3b). Permanent strain is accumulated rapidly during approximately the first 30 cycles of loading (up to 6 ns, 200 ps/cycle) and remains constant afterward. Further, when we performed additional equilibration for 10 ns on the processed system (originally equilibrated for 2.5 ns), no increase of the dimensions of the system is observed (see Figure S3c). We infer that permanent strains cannot be associated with further relaxation of the system. The manifestation of permanent strains in the unloaded bilayer membrane reveals an underlying dissipative mechanism, which at this stage needs to be clarified. The dissipative mechanism could well be associated with parylene itself (e.g., plasticity) or the interface between parylene and graphene (e.g., slippage).

We begin our investigation by studying the evolution of the potential energy in the polymer (see Figure 3c). We observe two trends in its evolution: (i) the energy oscillates at the cycle frequency due to bond extension and relaxation, but (ii) also the energy decreases globally after each cycle of loading. Similarly to permanent strains, the potential energy decrease stops after 30 cycles of loading. The second observation implies a further relaxation of the polymer as cycles proceed. The potential energy decrease in the polymer does not tend to support that the accumulation of permanent strain is induced by a plastic flow within the polymer only.

We compute the average in-place stresses σ_*xx*_ and σ_*yy*_ in the graphene sheet right after the polymerization (step 2) and after the complete equilibration of the processed membrane (step 5) and before any cyclic loading (see Figure 3d). In the equilibrated system, we find that the graphene sheet hosts non-negligible residual in-plane axial stresses. These stresses were not present immediately after polymerization, which implies they are transferred during the relaxation from the out-of-equilibrium polymer. We conclude that at the end of the simulation of the complete process, residual self-equilibrated stresses coexist in the polymer and the graphene layers. The interface between the polymer under tension and the graphene under compression is therefore subject to a constant residual shear stress before any cyclic loading is applied.

**Figure 4:**
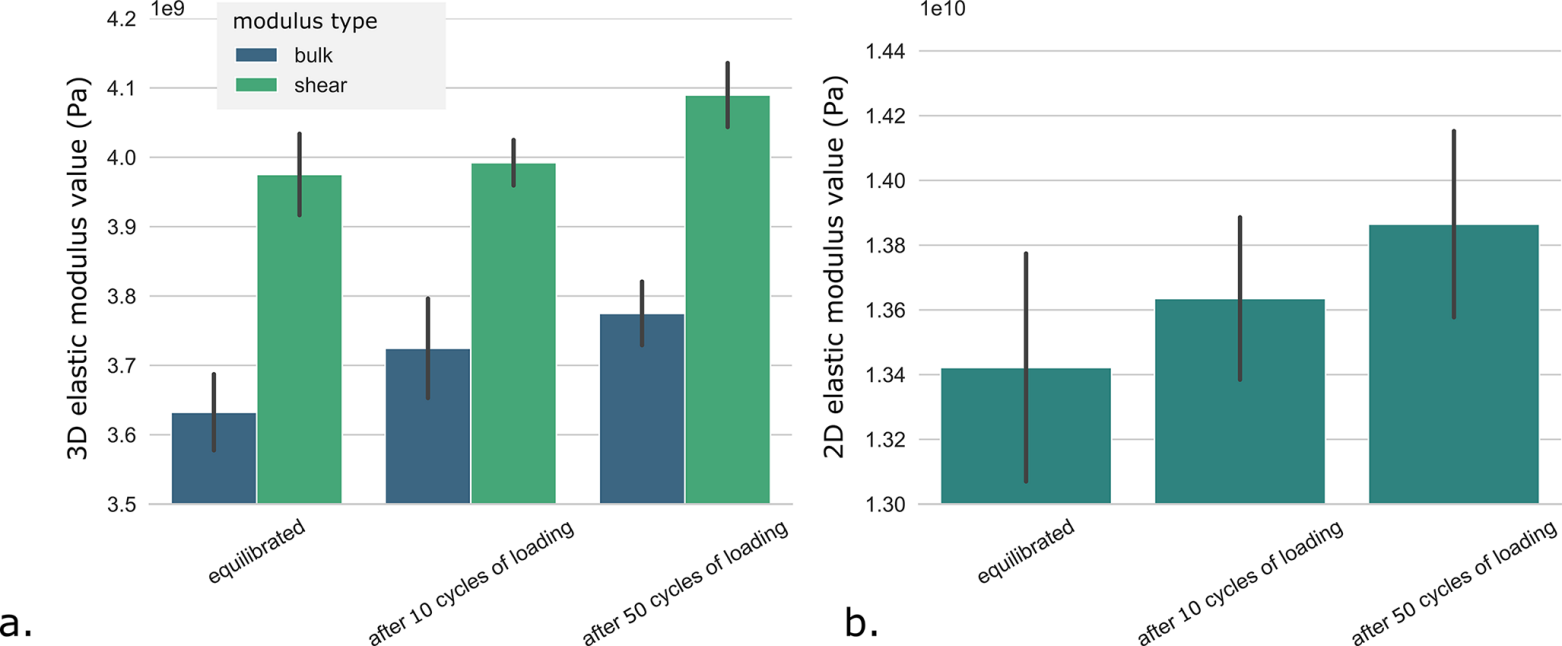
Cycles of loading alter elastic mechanical properties. Increase of (a) the 2D in-plane (*xx* or *yy*) elastic moduli as well as of (b) the 3D (bulk and shear) moduli with the number of applied cycles of loading. Elastic mechanical properties are computed after equilibration, after 10 cycles of loading, and after 50 cycles.

The decrease of potential energy in the parylene seen after multiple cycles of loading is associated with the release of the initial positive residual stresses within the polymer. This finding supports the emergence of a slippage mechanism at the interface between graphene and parylene induced by cycles of loading. The slippage mechanism is found to appear early on after the first high-frequency acoustic waves effect are felt on the bilayer membrane. The mechanism continuously alters the effective mechanical properties of the nanocomposite during first use cycles (see Figure 4). We observe an increase of the 2D (in-plane, *xx* or *yy*) and 3D (bulk and shear) elastic moduli of the bilayer with the number of cycles of loading. Reduction in internal stresses causes parylene and graphene to be in the same mechanical state, that is, simultaneously in tension or in compression. In turn, both materials tend to oppose one another more synchronously, enhancing the mechanical properties. Elastic mechanical properties are directly linked to the bending stiffness of the pressure sensor; therefore this needs to be taken into account during the calibration of the sensing device.

### A Pinning Mechanism Dependent upon Electrostatic Interactions

**Figure 5:**
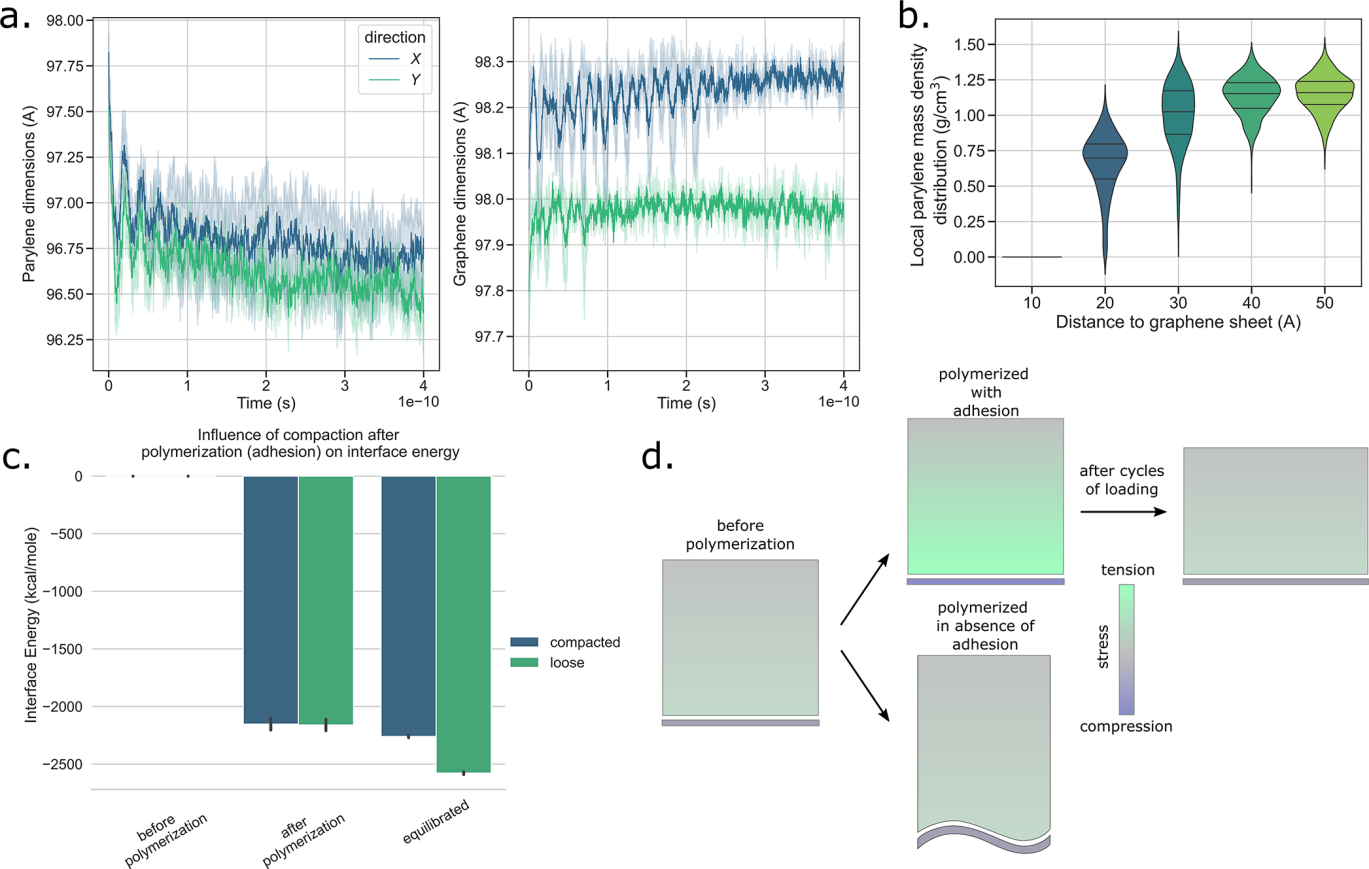
Electrostatic pinning causes self-equilibrated stresses during processing. (a) Time evolution of the in-plane dimensions of the remaining component after removal of the other; parylene shrinks and graphene expends. (b) Evolution of the distribution of the in-plane local density of the polymer with the distance of the plane from the graphene sheet. Local densities are computed from 100 sampling points in each plane. The width of the distributions shrink while moving away from graphene. (c) Influence of the compaction of the polymer onto graphene on the evolution of interface parylene–graphene energy between step 2 (after polymerization) and step 5 (after equilibration). (d) Schematization of the influence of adhesion between the polymer and the graphene sheet on the amplitude of self-equilibrated stresses. (e) The graphene sheet evolves into undulating patterns when parylene-C is replaced by parylene-F as the adhesion between the polymer and the sheet decreases.

The hypothesis of self-equilibrated residual stresses in the membrane constituents during processing by deposition–polymerization is further tested by removal of one of the two constituents (see Figure 5a). When we remove the graphene sheet, we immediately witness shrinking of the parylene layer in both in-plane dimensions. Conversely, when we remove the parylene layer, we observe expansion of the graphene sheet. These two virtual experiments reveal the underlying mechanism. During polymerization of parylene onto graphene, covalent bonds between parylene molecules are formed out-of-equilibrium. However, because of their deposition onto graphene, the interaction with the underlying sheet prevents their relaxation. The graphene sheet “pins” the polymer in a stretched state whose associated tensile stresses are balanced by compressive stresses in the graphene.

In order to further test the pinning mechanism hypothesis, we look for anomalous polymer behavior near the interface with graphene. In particular, we observe the distribution of polymer local density near the interface (see Figure 5b), data easily obtained from MD simulations. Out-of-equilibrium parylene, free from constraints, should relax toward a state of homogeneous density. We discretize the volume of parylene in 1 000 000 cells of 0.1 × 0.1 × 0.1 nm^3^, and for each cell we compute the local density of parylene. We study the distribution of these local densities in individual planes at constant distance from the graphene sheet. We find that the distributions tend to shrink as we move away from the sheet. That is, the local densities are more heterogeneous near the graphene and in particular below a 3 nm distance, which further supports the hypothesized pinning mechanism. The distributions appear to have converged beyond 4 nm.

We now modify the processing of the bilayer membrane to inspect the effect of contact surface area between parylene and graphene. More precisely, we build new bilayer membranes, but this time step 3 is removed as we do not compress the polymer onto graphene between polymerization and equilibration. We compare the interface energy between the two constituents, in the normal (compacted) and the modified (loose) processing conditions (see Figure 5c). We find that in loose conditions, the interface energy accumulated right after polymerization is able to relax more than in the compacted conditions. Indeed, the later conditions induce higher adhesion between parylene and graphene, which prevents slippage, density homogenization, and subsequently stress relaxation at their interface. The effect of compaction and adhesion on the hypothesized pinning mechanism is summarized in the drawing in Figure 5d. Besides, loose conditions are also representative of what could happen if larger graphene sheets were considered, for which curvature may play a more substantial role. Such large sheets were not considered here, since they remain out of reach with the supercomputers and allocations available to us at the time of writing.

Instead of removing the compaction step during the processing, we now modify the chemistry of the polymer. We employ a more polarized polymer, that is, parylene-F (see Figure 1a) with four fluorine atoms per monomer unit. Qualitatively, we observe that when replacing parylene-C by parylene-F, the graphene sheet develops ripples when the system relaxes after polymerization (see Figure 5e). The sheet appears to buckle. The replacement of the atoms bound to the benzene rings of parylene-C by fluorine is expected to reduce adhesion between graphene and parylene. The absence of hydrogen atoms and higher electronegativity alters van der Waals interactions, reducing hydrogen bonding and favoring parylene interactions with itself rather than with graphene. In turn, when the polymerized parylene relaxes and shrinks, the limited friction no longer enables the graphene sheet to pin the parylene out-of-equilibrium. The sheet starts to buckle under the in-plane compression induced by the shrinking volume because it does not remain stable in-plane when embedded by the fluorinated polymer. This demonstrates the potential for an alternative synthesis route of the nanocomposite which relies on parylene-F. The use of a more electronegative polymer alleviates the buildup of internal stresses during the processing.

## Conclusions

We have investigated the durability and mechanical stability of bilayer membrane nanocomposites comprised of parylene-C and graphene when they are employed as sensors and transducers by simulating processing and operating conditions using classical molecular dynamics. We found that the mechanical properties of the nanocomposite evolve rapidly during operation when subjected to stretching cycles corresponding to the effect of sensed acoustic waves. By unraveling the underlying mechanism, we proposed a solution to achieve mechanical stability. In particular, the nanocomposite accumulates a permanent strain rapidly after the onset of external loading. We discovered that the permanent strain is the consequence of slippage at the interface between the membrane constituents. We found that slippage is due to the release of elastic energy stored as residual interfacial stresses in the polymer and the graphene layers, akin to a pinning mechanism that arises during the deposition–polymerization process. Due to this pinning mechanism, graphene maintains the polymer in an out-of-equilibrium state, resulting in non-negligible internal tensile stresses. We identified the dependence of these stresses upon adhesion and electrostatic forces acting between the parylene-C matrix and graphene. The pinning mechanism, the associated interfacial stresses, and their subsequent relaxation upon operation are detrimental to the durability and accuracy of capacitive micromachined ultrasound transducers and to nanoelectromechanical systems in general. To mitigate these issues, we recommend the use of polymer dielectrics displaying low electrostatic interactions with graphene to relieve the interfacial stresses and ensure mechanical stability. Our findings demonstrate the need to carefully select the chemical composition of these membranes for the future design of high-accuracy and reliable pressure-sensing devices based on ultrathin layered polymer–graphene nanocomposites.

## Data Availability

The data that support the findings of this study are available from the corresponding author upon reasonable request.

## Supporting Information Available

Validation of the mechanical properties of the computational molecular model for parylene-C (bulk modulus, glass transition temperature); validation of the applied strain rate; convergence of predicted elongation with decreasing rate (PDF)

## Notes

**Code Availability.** The Polymatic code (https://nanohub.org/resources/17278) used to build parylene models and the code to build graphene models (https://github.com/velocirobbie/make-graphitics/) are fully available online.
