# Supplementary material for: Mechanically Stable Ultrathin Layered Graphene Nanocomposites Alleviate Residual Interfacial Stresses: Implications for Nanoelectromechanical Systems
Source: ACS Appl Nano Mater. 2022 Dec 14;5(12):17969–76. doi: 10.1021/acsanm.2c03955 (PMC9791614; doi:10.1021/acsanm.2c03955)
Supplement: Supplementary file 1 — an2c03955_si_001.pdf [file an2c03955_si_001.pdf]

Supporting Information

-

Mechanically-Stable Ultra-Thin Layered Graphene Nanocomposites Alleviate Residual Interfacial Stresses: Implications for Nanoelectromechanical Systems

Maxime Vassaux,[†,‡] Werner M. Roa,[‡] James L. Suter,[‡] Aravind Vijayaraghavan,[¶] and Peter V. Coveney[*,‡,§,‖]

†*Univ. Rennes, CNRS, IPR (Institut de Physique de Rennes) - UMR 6251, Rennes, 35000, France*

‡*Centre for Computational Science, Department of Chemistry, University College London, London, WC1H 0AJ, United Kingdom*

¶*Department of Materials and National Graphene Institute, The University of Manchester, Manchester, M13 9PL, United Kingdom*

§*Advanced Research Computing Centre, University College London, WC1H 0AJ, United Kingdom*

‖*Informatics Institute, University of Amsterdam, Amsterdam, 1098 XH, The Netherlands*

E-mail: p.v.coveney@ucl.ac.uk

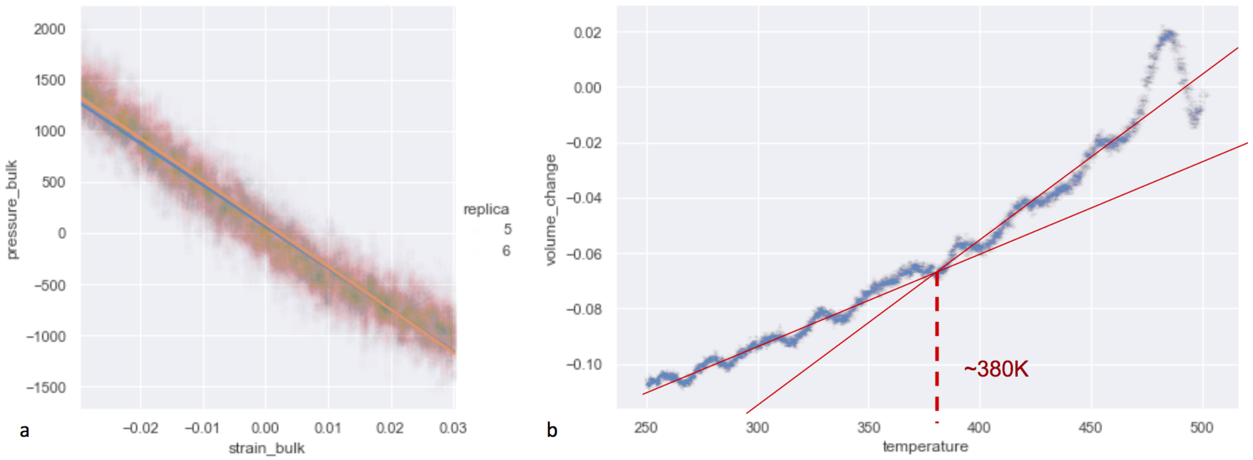

Figure S1: Validation of the parylene C molecular model. (a) The mean of the bulk modulus is 4.19 +/- 0.01 GPa and the mean of the Young's modulus is 2.52 +/- 0.01 GPa. (b) Simulations are also able to recover the elastic modulus and the glass temperature transition of the polymer.

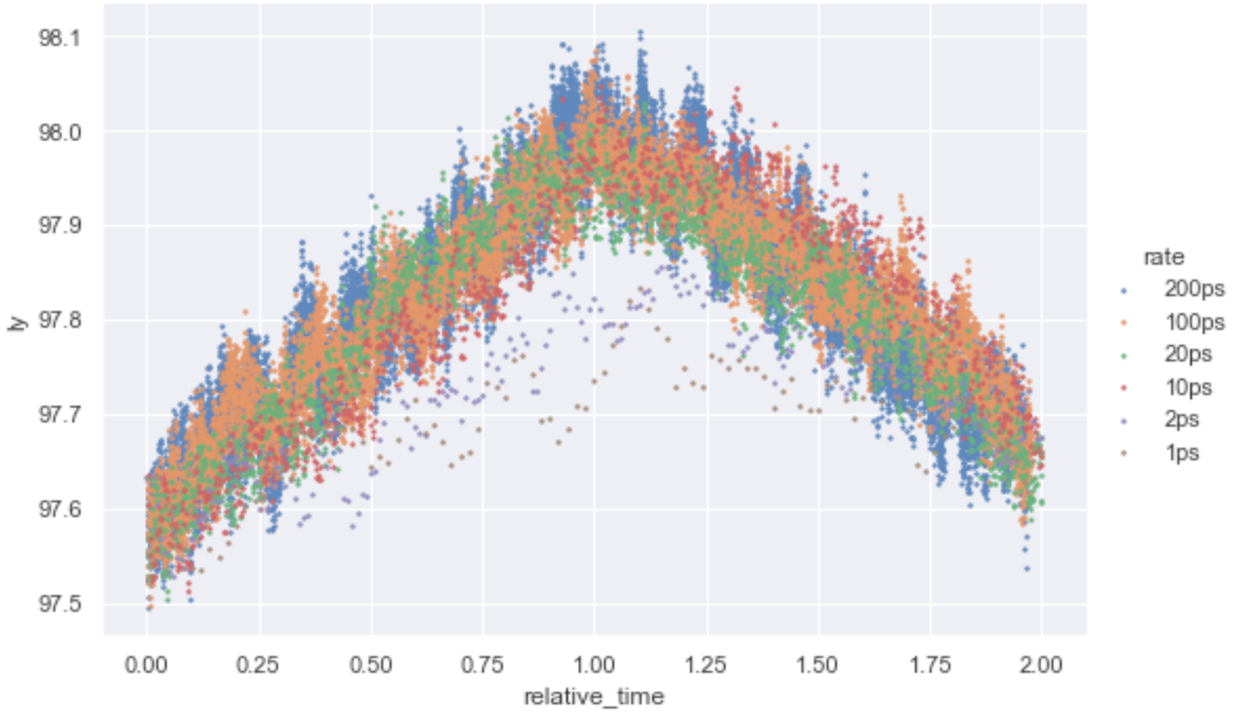

Figure S2: Convergence of the mechanical response with the rate of loading confirming the time scale separation. At cycles duration beyond 100 ps, the deformation amplitude of the membrane appears to converge. This suggests that 100 ps is the shortest duration admissible to complete a cycle of loading while preserving strain rate independence.

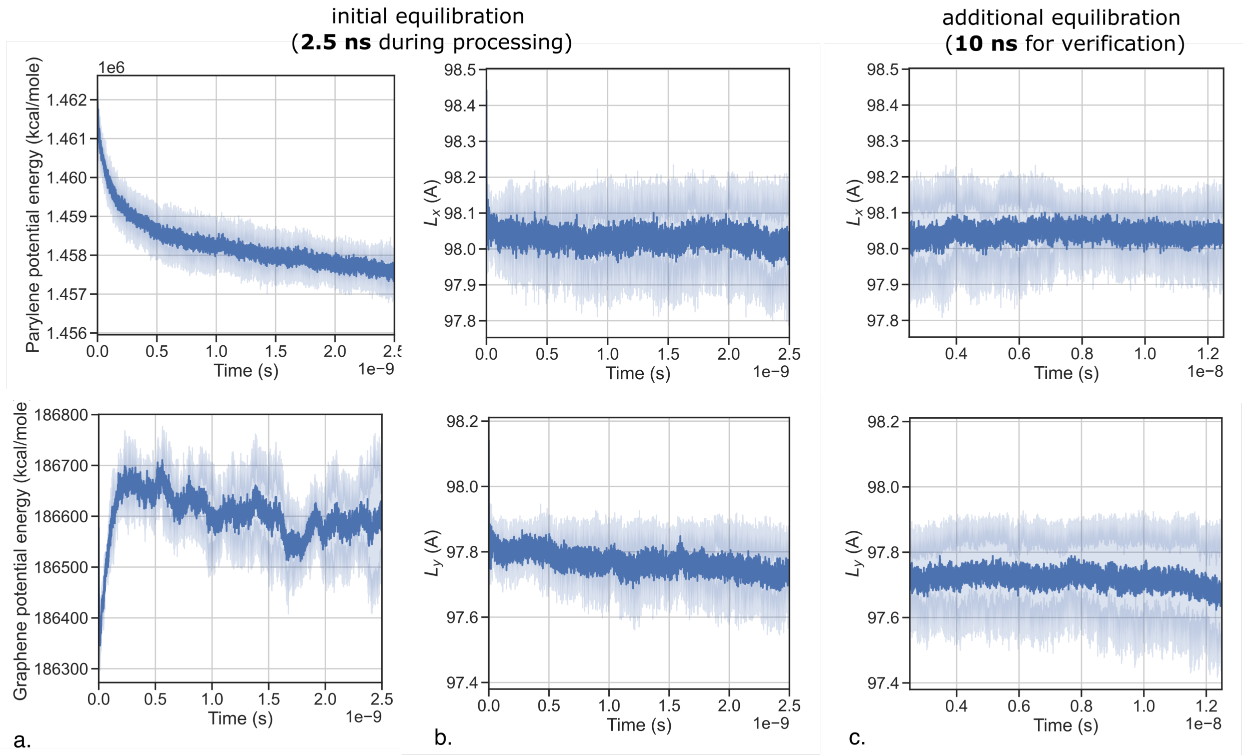

Figure S3: Verification of the parylene-C equilibrium. (a) Evolution of the constituents potential energies (parylene and graphene) and (b) the lateral dimensions (L_x and L_y) of the molecular system during the 2.5 nanosecond of equilibration for the processing. From 1.5 ns to 2.5 ns, the potential energy of graphene is stable, while the potential energy of parylene-C varies by less than 0.1%, therefore the system is assumed to be relaxed. (c) Additional equilibration is performed during 10 ns to verify that the evolution of the system observed during testing is not induced by further relaxation. No increase in systems dimensions is observed during the additional relaxation.
